# Immunological and Genetic Investigation of SARS-CoV-2 Reinfection in an Otherwise Healthy, Young Marine Recruit

**DOI:** 10.3390/pathogens10121589

**Published:** 2021-12-08

**Authors:** Andrew G. Letizia, Catherine E. Arnold, Bishwo N. Adhikari, Logan J. Voegtly, Lindsay Glang, Gregory K. Rice, Carl W. Goforth, Megan A. Schilling, Dawn L. Weir, Francisco Malagon, Irene Ramos, Sindhu Vangeti, Ana S. Gonzalez-Reiche, Regina Z. Cer, Stuart C. Sealfon, Harm van Bakel, Kimberly A. Bishop-Lilly

**Affiliations:** 1 Infectious Disease Directorate, Naval Medical Research Center, Silver Spring, MD 20910, USA; andrew.g.letizia.mil@mail.mil (A.G.L.); carl.w.goforth.mil@mail.mil (C.W.G.); megan.a.schilling.mil@mail.mil (M.A.S.); dawn.l.weir.mil@mail.mil (D.L.W.); 2 Defense Threat Reduction Agency, Fort Belvoir, VA 22060, USA; catherine.e.arnold13.civ@mail.mil (C.E.A.); bishwo.n.adhikari@usda.gov (B.N.A.); 3 Genomics and Bioinformatics Department, Biological Defense Research Directorate, Naval Medical Research Center–Frederick, Fort Detrick, MD 21702, USA; logan.j.voegtly.ctr@mail.mil (L.J.V.); lindsay.a.glang.ctr@mail.mil (L.G.); gregory.k.rice.ctr@mail.mil (G.K.R.); francisco.malagonbautista.ctr@mail.mil (F.M.); regina.z.cer.civ@mail.mil (R.Z.C.); 4 Leidos, Inc., Reston, VA 20190, USA; 5 Department of Neurology, Icahn School of Medicine at Mount Sinai, New York, NY 10029, USA; irene.ramos-lopez@mssm.edu (I.R.); sindhu.vangeti@mssm.edu (S.V.); stuart.sealfon@mssm.edu (S.C.S.); 6 Department of Genetics and Genomic Sciences, Icahn Institute for Data Science and Genomic Technology at Mount Sinai, New York, NY 10029, USA; anasilvia.gonzalez-reiche@mssm.edu (A.S.G.-R.); harm.vanbakel@mssm.edu (H.v.B.)

**Keywords:** SARS-CoV-2, reinfection, COVID-19, CHARM, next generation sequencing (NGS), genomics, marines

## Abstract

We used epidemiologic and viral genetic information to identify a case of likely reinfection in an otherwise healthy, young Marine recruit enrolled in the prospective, longitudinal COVID-19 Health Action Response for Marines (CHARM) study, and we paired these findings with serological studies. This participant had a positive RT-PCR to SARS-CoV-2 upon routine sampling on study day 7, although he was asymptomatic at that time. He cleared the infection within seven days. On study day 46, he had developed symptoms consistent with COVID-19 and tested positive by RT-PCR for SARS-CoV-2 again. Viral whole genome sequencing was conducted from nares swabs at multiple time points. The day 7 sample was determined to be lineage B.1.340, whereas both the day 46 and day 49 samples were B.1.1. The first positive result for anti-SARS-CoV-2 IgM serology was collected on day 49 and for IgG on day 91. This case appears most consistent with a reinfection event. Our investigation into this case is unique in that we compared sequence data from more than just paired specimens, and we also assayed for immune response after both the initial infection and the later reinfection. These data demonstrate that individuals who have experienced an infection with SARS-CoV-2 may fail to generate effective or long-lasting immunity, similar to endemic human beta coronaviruses.

## 1. Introduction

Despite relatedness to SARS-CoV, the virus that caused an outbreak in China in 2003, and our prior knowledge of many other beta coronaviruses routinely circulating, such as 229E, NL63, HKU1, and OC43, SARS-CoV-2 continues to present significant diagnostic and medical surveillance challenges. It is known that coronavirus reinfection is common, sometimes even within a given cold and flu season [1,2,3]. However, until recently, there has never been such widespread deployment of viral nucleic acid detection and sequencing, so the natural length of time for shedding a given virus’s nucleic acids is not well known, nor do we know how or whether this would vary between an asymptomatic or a symptomatic case.

For a newly emergent virus such as SARS-CoV-2, without time to conduct the full range of scientific study necessary to give a complete understanding of infection dynamics, strain level cross-protection, and the durability of naturally acquired immunity, it can be difficult to discern between true reinfection versus a long-standing or recrudescent infection. It is also unknown how long an individual is infectious versus shedding viral nucleic acid fragments in the absence of infectious virus particles. The U.S. Centers for Disease Control and Prevention (CDC) noted this current lack of information in their guidance entitled, Criteria for Investigating Suspected SARS-CoV-2 Reinfection, updated in October of 2020 [4].

We present an individual case of SARS-CoV-2 reinfection from the COVID-19 Health Action Response for Marines (CHARM) study—in a young, otherwise healthy United States Marine recruit with an initial asymptomatic infection and a subsequent symptomatic reinfection. Viral genome sequencing and serology were conducted at multiple time points, including asymptomatic, symptomatic, and reverse transcription polymerase chain reaction (RT-PCR)-negative time points, and CDC guidelines for investigation of reinfection were applied. Taken as a whole, the data are most consistent with a case of reinfection. We present these data and discuss the caveats and public health implications.

## 2. Results

### 2.1. Clinical Presentation and SARS-CoV-2 Sampling

**Figure 1:**
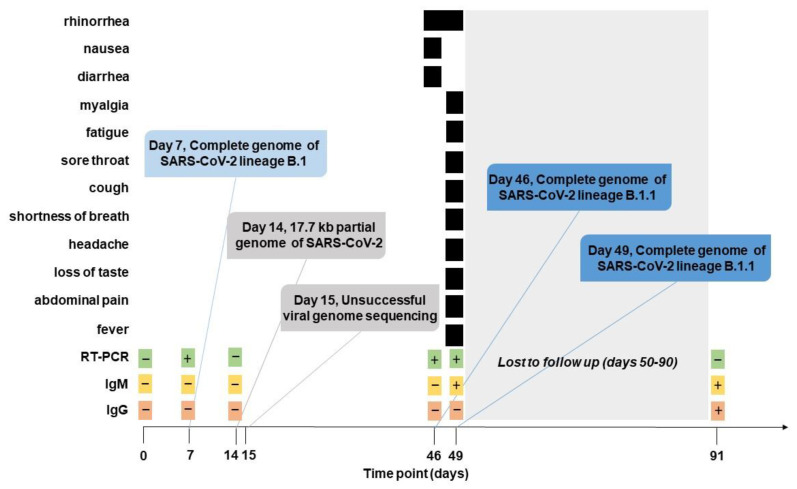
Timeline of reinfection. Plus (+) or minus (−) in green boxes indicate the results of SARS-CoV-2 RT-PCR testing, in yellow boxes they indicate the results of IgM testing, and in peach boxes they indicate the results of IgG testing. Black bars indicate the days on which the patient reported each symptom.

The participant was an 18-year-old male who was unvaccinated without any significant past medical history and specifically denied multiple infections as a child or evaluation for immunodeficiency disorders. He denied a history of smoking or asthma. The participant self-quarantined at home for 14 days without exposure to a sick contact prior to arrival and had a negative RT-PCR test as well as no detectable IgM and IgG antibodies to SARS-CoV-2 upon enrollment into the study (at day 0) on June 17, 2020. However, he did have a positive RT-PCR to SARS-CoV-2 upon routine sampling on day 7 as part of the prospective study. At this time, he reported no symptoms. RT-PCR testing at study day 14 was negative and he continued to be asymptomatic. He did not follow up as scheduled with study personnel, but on day 46, he reported to medical personnel with rhinorrhea, nausea, and diarrhea. SARS-CoV-2 RT-PCR testing as part of the study was positive at that time. His RT-PCR result remained positive on study day 49 and although his diarrhea and nausea had resolved, he had a fever and reported myalgia, fatigue, rhinorrhea, sore throat, cough, shortness of breath, headache, loss of taste, and abdominal pain. IgG antibody levels in serum to SARS-CoV-2 were negative at days 7 and 14, and remained negative at days 46 and 49, indicating the absence of the development of a long-lasting humoral immune response after the first infection at day 7. IgM antibodies were negative at days 7, 14, and 46, and were detected by the first time at day 49 (titer 450) as a response to the second infection (day 46). After study day 49, the participant did not follow up for the next six weeks, and therefore, it is unknown how long he remained RT-PCR positive for SARS-CoV-2 or how long his symptoms persisted. His only other follow-up was at study day 91, by which time all symptoms had resolved and his RT-PCR result for SARS-CoV-2 was negative, but presented high IgG (titer 12150) and IgM (titer 1350) antibody levels. A summary of the course of infection and relevant findings is presented in Figure 1.

### 2.2. Virus Characterization

Whole genome sequencing was performed from day 0, 7, 14, 15, 46, and 49 samples. The genomes were sequenced and analyzed independently in two different laboratories and the resulting sequences were validated against each other. Not surprisingly, only the RT-PCR positive time points (days 7, 46, and 49) yielded full viral genomes. However, a partial genome, a 17.7 kb consensus sequence, was obtained from the day 14 sample, which was an RT-PCR negative sample. The day 7 sample required multiple sequencing reactions in both laboratories in order to yield a high-quality dataset, likely due to less viral material at that time point, consistent with higher Ct values at day 7 (values over 28 for all three targets; Table 1).

**Figure 2:**
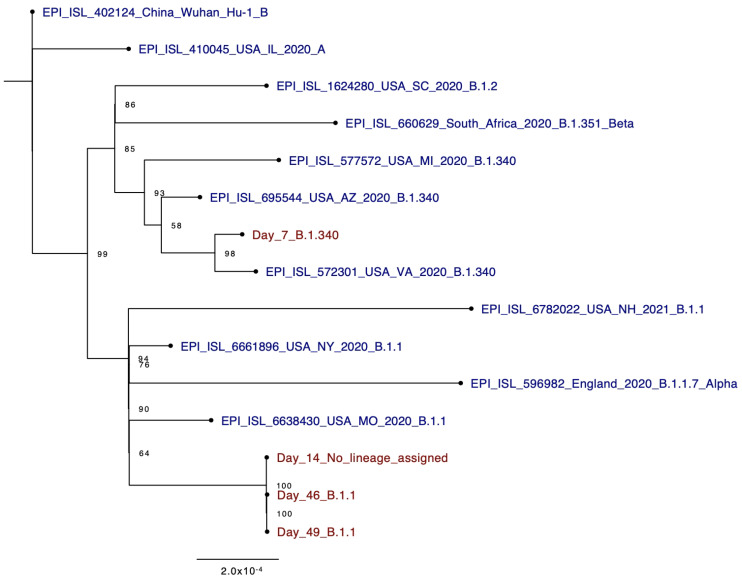
Phylogenetic tree of consensus genomes with GISAID representatives. Maximum Likelihood Phylogeny tree shown with the four timepoint sequences represented in dark red (Day_7, Day_14, Day_46, and Day_49) and 11 GISAID representative sequences in dark blue. Lineage assignments by Pangolin v3.1.16 included for all sequences. Bootstrap values are reported at nodes.

Phylogenetic analysis and variant calling were conducted for day 7, 46, and 49 genomes and the data were assessed in accordance with CDC criteria for genetic evidence of reinfection. Overall, the day 46 and day 49 genomes were more similar to each other than to the day 7 genome (Figure 2). Due to incompleteness, the 17.7 kb consensus sequence obtained from day 14 could not confidently be assigned a lineage. However, the 17.7 kb sequence was slightly more similar to the day 46 and day 49 genomes than to the day 7 genome. Whereas the day 7 sample was determined to be lineage B.1.340/Clade 20C, both the day 46 and day 49 samples were determined to be lineage B.1.1/Clade 20B. The B.1.1 lineage is differentiated from B.1.340 in part on the basis of three adjacent single nucleotide variations (SNVs), collectively represented as 28,881-28,883 GGG>AAC (N: R203K, G204R). At that genome position, which in part differentiates B.1.340 from B.1.1, the day 14 genome was only covered at 4× depth (too low for consensus genome calling by iVar). Extraction of the consensus sequence without any coverage threshold revealed AAC in those positions, which is consistent with B.1.1, similar to the day 46 and day 49 samples. SNVs characteristic to B.1.340 and not B.1.1 were present in the day 7 genome, including C1059T (ORF1ab: T265I), A4197G (ORF1ab: E1311G), G25563T (ORF3a: Q57H), and A28715T (N:T148S). The day 14 consensus genome did not have G25563T (ORF3a: Q57H) and the other positions did not have adequate coverage for consensus calling by iVar, but extraction of the of the consensus sequence without any coverage threshold revealed the reference alleles at those positions, consistent with B.1.1. Because it was only a partial genome sequence, the day 14 genome was not used in downstream analyses.

As compared to the reference, four SNVs were common to all three complete genomes (days 7, 46, and 49): C241T, C3037T, C14408T, and A23403G. Despite multiple library preparation and sequencing attempts in both laboratories, the day 7 data contained overall many more variations, most of which were present at relatively low concordance, characteristics not uncommon to samples with low viral load. SNV profiles for this sample were compared to other samples sequenced at the same time and no conclusive provenance of sample bleed-through could be traced. Specifically, of the 53 SNVs unique to the day 7 sample as compared to days 46 and 49, thirty SNVs were present at less than 10% frequency (Table 2). Five SNVs were unique to day 46, but all were present at less than 5% frequency and all were in ORF1ab. Four out of five of those were nonsynonymous SNVs in the portion of ORF1ab that encodes for nsp16 and in close proximity to each other; the other was a silent mutation in the portion of ORF1ab that encodes nsp2 (Table 3). Twenty-five SNVs were unique to the day 49 sample, and all of these were present at less than consensus level frequency, with 13 of 25 present at less than 10% frequency. Most of these were in ORF1ab, but there were also five in the spike gene, two in ORF7a, one in ORF7b, and three in the gene encoding nucleocapsid phosphoprotein (Table 4).

**Figure 3:**
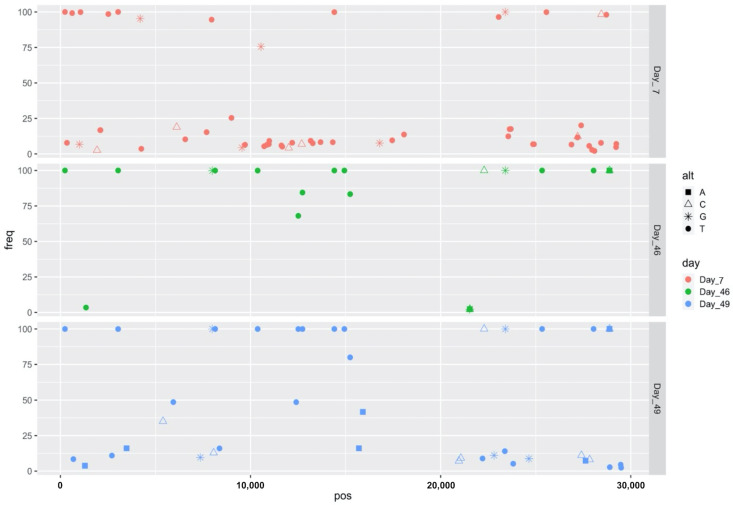
SARS-CoV-2 single nucleotide variations (with respect to Wuhan reference genome NC_045512.2) and frequency over time. SARS-CoV-2 genome coordinates are plotted on the X-axis and allele frequencies on the Y-axis. Alternative alleles of SNVs are represented by different shapes.

Overall, the proportion of high concordance SNVs increased over time (Figure 3). There were 42 SNVs shared between days 46 and 49. Both days 46 and 49 shared the same four SNVs with day 7. Days 46 and 49 contained a greater number of high-frequency SNVs as compared to day 7.

## 3. Discussion

Herein we present an investigation into a probable SARS-CoV-2 reinfection in a young Marine recruit without a history of immunodeficiency, asthma, or smoking. The participant was asymptomatic and RT-PCR positive at day 7 of the CHARM study, then became RT-PCR negative by day 14 until day 46, whereupon he reported symptoms consistent with COVID-19 and the diagnosis was confirmed by RT-PCR testing. Our investigation into this case is unique in that we compared sequence data from more than just paired specimens, and we also assayed for immune response after both the initial infection and the later reinfection.

Generally speaking, high-throughput viral amplicon sequencing is much more sensitive than unbiased metagenomic sequencing, due to multiple rounds of viral genome amplification with specific primers prior to sequencing library construction and deep sequencing, and thus, amplicon sequencing is often robust enough to generate viral genome sequences directly from complex clinical samples such as serum even with relatively high Ct values, such as Ct = 29 [5]. Therefore, sequencing was attempted on all available samples from the patient, including two time points that were negative by the diagnostic RT-PCR assay that was used (days 0 and 14), three time points that were positive (days 7, 46, and 49), and one that had not been tested by RT-PCR (day 15). Not surprisingly, samples with lower Ct values (an indicator of higher viral load) performed better than the sample with the higher Ct values (day 7), although this day 7 sample was only just slightly over what is viewed as a good Ct cutoff for sequencing (a Ct value of 28 as per Jacot et al. [6]). The day 14 sample, which was negative by diagnostic RT-PCR, yielded a partial genome sequence, which could be consistent with the process of clearance of the infection seven days post the initial RT-PCR-positive result and fragments of viral genomic material still in circulation. Given the above-mentioned sensitivity of viral amplicon sequencing, it is not impossible to detect partial viral genetic sequences from RT-PCR-negative time points. The RT-PCR assays only characterize the presence/absence of specific targets within three specific genes, and the results can, therefore, be easily affected by antigenic drift and shift as well as other means of missing targets, such as deletions or partial degradation of nucleic acids. By contrast, there are many more targets available over the entire virus genome for the many pairs of viral amplicon sequencing primers to bind. In other words, when titer is low and/or viral genetic material is incomplete, reliance on only three targets might be less sensitive to identify the presence of SARS-CoV-2-specific RNA than detection based on, essentially, any part of the entire genome (220 and 98 amplicon targets for YouSeq and ARTICv2, respectively). In fact, successful SARS-CoV-2 genome sequencing from an RT-PCR-negative time point has been previously reported in at least one case [7].

The participant did not develop a detectable humoral response upon the first infection, which explains the negative IgM and IgG response before days 49 and 91, respectively. While this scenario is not common, several reports have shown various percentages of lack of seroconversion after SARS-CoV-2 infection in healthy immunocompetent adults [8,9,10]. Younger age among adults and high Ct values, consistent with the participant’s age and the high Ct values at the day 7 infection, has been associated with a higher probability of not developing SARS-CoV-2 antibodies [9]. A robust humoral immune response was developed after the second infection (day 46), which was characterized by higher viral load and presence of symptoms. The second infection was detected for the first time at day 46, when the participant first reported symptoms (Figure 1). Antibody responses take a few days to be established and detected, and IgM responses are often detected earlier than IgG responses [11,12]. Here, the IgM response was detected for the first time at day 49 (3 days after second infection), and the IgG response was not detectable at day 49, likely because it was still too low to be detected. However, the next available time point at day 91 showed presence of IgG antibodies, indicating that the second infection had promoted production of IgG antibodies, although its levels were not detectable during the first 3 days post first RT-PCR positive time points. The dynamics of the antibody response after the second infection are in line with previous reports [11,12]. Our results here suggest that serological responses to asymptomatic or mild infections bear further investigation in order to aid our understanding of how previous infection with SARS-CoV-2 may or may not protect against future infection.

Early in the COVID-19 pandemic, the parameters used for investigation of apparent reinfection cases varied somewhat from investigator to investigator [13,14,15]. Now, the CDC guidance provides a useful, standardized framework with which investigators can work to produce comparable datasets and provide acceptable evidence for conclusions. In this guidance, the CDC proposed using two types of criteria to investigate reinfections—1) investigative criteria aimed at identifying cases with a higher index of suspicion for reinfection and 2) viral genetic data from paired samples. In addition to proposing two types of criteria, the CDC went further to provide specific recommendations for quality of sequencing data and the degree of difference(s) detected in genetic data, based on SNVs, lineage calls, and the viral mutation rate. These guidelines are a helpful step toward establishing a definition of SARS-CoV-2 reinfection and how it is determined. It is very possible that as more data are collected, definitions and guidelines will continue to evolve.

In their guidance, updated in October of 2020 [4], the CDC proposed that cases within two specific windows of time be investigated—90 days or more after initial infection/illness or 45–89 days from initial illness, the latter only if the individual is symptomatic the second time. This case of SARS-CoV-2 reinfection was in an otherwise healthy, young individual who was asymptomatic the first time and symptomatic the second time, with the reinfection occurring very close to the CDC-recommended window that should be investigated (39 days versus the recommended 45 days). Secondly, regarding investigative criteria to identify cases with a higher index of suspicion for reinfection, this was not an individual who was staying home or working in a socially-distanced environment. Instead, this active duty military participant was training in a close-quarters setting that requires frequent, close contact and was known to carry a high risk of infection by SARS-CoV-2 [16].

Furthermore, this case is backed with genetic evidence that mostly fulfills the CDC’s Investigative Criteria for Suspected Cases of SARS-CoV-2 Reinfection. Those criteria state that SNV analysis alone is not sufficient to declare reinfection rather than long-term infection and that distinct lineages of virus is better evidence. The CDC recommends paired respiratory specimens from the individual be sequenced; in this case, due to CHARM’s study design, we had more than two samples to assay genetically. The CDC advises that high-fidelity sequencing platforms (Q score per read >30) be used for consensus sequence generation and that amplicon primer sequences be removed from the genome assembly, both of which were done in this study. CDC also recommends a genome coverage >100/per base position, Q score of consensus >30 with 99% of the genome covered, and 1000× average genome coverage for analysis of minor variation. Although all the samples were relatively deeply sequenced, whereas only the day 46 sample ultimately met the criteria of genome coverage >100/per base position, both the day 7 and day 46 samples met the criteria of Q score of consensus >30 with 99% of the genome covered, and both the day 46 and day 49 samples met the criteria of 1000× average genome coverage for analysis of minor variation. Additionally, although the day 14 sample resulted in only roughly two thirds of the virus genome being covered, we were able to use those data in a limited capacity as well. The CDC advises that if low fidelity sequencing platforms (Q score per read <30) are used, verification of SNVs via alternate sequencing method is conducted. At the beginning of the study, some Ion Torrent data were produced, and compared to Illumina data as well, but due to the associated Ion data quality scores, only Illumina data are reported herein, with the exception of small portions of the day 49 genome that were filled in using Ion reads.

The day 7 sample was found to have the most unique SNVs of the three time points with full genome data. The four SNVs shared between day 7 and later time points are well-documented SNVs found in SARS-CoV-2 genomes. The higher proportion of low frequency unique mutations on day 7 supports an isolated case of early infection. Most of the SNVs in the day 46 dataset are shared with day 49. The proportion of high frequency SNVs is higher in the day 49 dataset than in the earlier time points, possibly suggesting viral adaptation over time. However, taken as a whole with both the circumstances of the individual and the genetic data encompassing SNVs and differing lineages, this case appears most consistent with reinfection. The caveat is that the day 7 sample produced data of lesser quality than would be optimal.

This case, for which we sequenced more than just paired samples, demonstrates the current challenges that are inherent to the investigation and adds to our understanding of SARS-CoV-2 reinfections. The relative risk of reinfection has been estimated from prospective RT-PCR and serological studies to be about one-fifth of those never infected [16,17]. This case demonstrates the possibility of reinfection even within a short timeframe while exposed to a high-risk congregant setting, such as basic training, associated with an absence of detectable serological response from the first infection. This case highlights that although an individual experienced natural infection, protective immunity cannot be assumed, and we demonstrate here that symptomatic reinfection within five weeks is possible. Continued public health education and vigilance is required as the medical and scientific communities collect data that will help provide a deeper understanding of SARS-CoV-2 infection and immunity.

## 4. Materials and Methods

### 4.1. The Study

The COVID-19 Health Action Response for Marines (CHARM) study has been previously described [18,19], but in brief, U.S. marine recruits were quarantined for two weeks prior to basic training, and within 48 h of arrival, offered the opportunity to volunteer in this longitudinal, prospective study. The study protocol was approved by the Naval Medical Research Center Institutional Review Board in compliance with all applicable federal regulations governing protection of human subjects. All participants provided written informed consent for participation. On day 0, mid-turbinate nares swabs collected in virus transport media (VTM) were assessed for SARS-CoV-2 by real-time reverse transcriptase polymerase chain reaction (RT-RT-PCR), with additional swabs assessed at study days 7, 14, 28, 42, and 56 for all participants using the FDA Emergency Use Authorization (EUA) TaqPath COVID-19 Combo Kit (Thermo Fisher Scientific; Waltham, MA, USA). If positive, more intensive sampling was performed twice per week for the first two weeks and then biweekly thereafter for the subsequent six weeks. Serum samples were also collected at the same time points, and presence of SARS-CoV-2-specific IgG and IgM antibodies was determined by ELISA.

### 4.2. SARS-CoV-2 Whole Genome Sequencing

RNA was extracted from VTM using TRIzol LS reagent (Invitrogen; Carlsbad, CA, USA) and used in both the ARTIC nCoV-2019 Sequencing protocol (v1) [20] and the YouSeq SARS-CoV-2 Coronavirus NGS Library prep kit (YouSeq; Winchester, UK). Approximately 100 ng of RNA was reverse-transcribed as in the protocol; however, the YouSeq reverse transcriptase was replaced with SuperScript IV (ThermoFisher Scientific; Waltham, MA, USA). cDNA was amplified using multiplex RT-PCR and either the associated ARTIC primer pools (v3 primers) or YouSeq primer pools. ARTIC amplicons were purified using 1× AMPure XP beads (Beckman Coulter; Indianapolis, IN, USA) and resuspended in nuclease free molecular grade water. Samples were then processed following the QiaSeq FX protocol (Qiagen; Valencia, CA, USA). Libraries were quality-checked using an Agilent High Sensitivity DNA kit (Agilent Technologies; Santa Clara, CA, USA) and quantitated using the Qubit DNA High Sensitivity assay (ThermoFisher Scientific) prior to sequencing using Illumina MiSeq v3 2x300 chemistry (Illumina; San Diego, CA, USA).

### 4.3. Bioinformatic Analyses

The Viral Amplicon Illumina Workflow (VIAW) was used to collate SARS-CoV-2 consensus genomes from the resulting sequence data (https://hub.docker.com/r/bdrdgenomics/viral_amplicon_illumina_workflow [21], accessed on 6 October 2020). Briefly, Illumina reads were quality trimmed and filtered to Q30 and minimum length of 50 bp using bbduk. Paired reads were merged using bbmerge with default settings [22]. Trimmed, filtered, and merged reads were aligned to the Wuhan reference genome (NCBI GenBank accession NC_045512.2/MN908947.3) using bbmap, v38.79, with local alignment and maximum insertion/deletion of 500 bp [23]. Primers were trimmed from sequences using align_trim from ARTIC workflow/pipeline, v 1.0.0 [24]. Once a high quality consensus genome was obtained, Single Nucleotide Variants (SNVs) were determined using SAMtools mpileup [25] and iVar (intrahost variant analysis of replicates) [26], using a minimum frequency of 0.3 and a minimum read depth of 10. In addition, low frequency SNVs were identified using a minimum frequency of 0.02, minimum alternate allele read depth of 15, and Phred score ≥ Q30 and visualized using the ggplot2 package in R (v3.6.3). Lineages were determined using Pangolin (Phylogenetic Assignment of Named Global Outbreak LINeages; v.3.1.16; https://github.com/cov-lineages/pangolin, accessed on 9 November 2021) [27]. Viral genome data are available in NCBI GenBank, accessions MW729373-MW729375 and raw sequencing reads are available in SRA, accessions SRR17073935- SRR17073938 (Supplementary Table S2).

For phylogenetic tree creation, reference sequences in fasta format were obtained from GISAID (www.gisaid.org, accessed on 29 November 2021) for representative lineages (see Supplementary Table S1), by carefully selecting only sequences that are considered complete and high coverage by GISAID. Phylogenetic tree was generated with MAFFT v7.487 [28] and IQ-TREE v2.1.4 [29] Maximum Likelihood Phylogeny GTR+G with 100 bootstrap replicates and visualized using FigTree v1.4.4.

### 4.4. SARS-CoV-2 Enzyme-Linked Immunosorbent Assay (ELISA)

The presence and levels of IgG and IgM SARS-CoV-2-specific antibodies in serum were determined using an enzyme-linked immunosorbent assay (ELISA), as previously described [16,18]. ELISA plates were coated with recombinant his-tagged receptor binding domain (RBD) (SinoBiological; Beijing, China) or trimerization-stabilized spike (S) protein (LakePharma; Irving, TX, USA). Serum samples were screened at a 1:50 dilution with RBD. Samples with an OD 492 nm value higher than the average of the negative controls (eight negative control sera collected before July 2019, Biochemed Services; Winchester VA, USA) plus three times their standard deviation (SD) in the screening assay underwent titration assay (six serial 1:3 serum dilutions starting at 1:50) using S protein. Serum samples were considered positive when the RBD screening assay and at least two consecutive dilutions in the S titration showed a higher OD 492 nm than the average of the negative controls plus three times their SD at the corresponding dilution, or 0.15 OD 492 nM.

## Figures and Tables

**Table 1:** Results of RT-PCR testing.

	Ct Value per Target			Patient Status
Time point (day)	S gene	N gene	ORF1ab	
7	28.9	28.3	28.5	Asymptomatic
46	16.9	16.8	17.4	Symptomatic
49	28.0	27.0	27.3	Symptomatic

**Table 2:** Fifty-three SNVs unique to the day 7 sample.

SNV	Frequency (%)	SNV Type	AA Change	Gene
C346T	7.83	synonymous	-	ORF1ab/nsp1
C619T	99.19	synonymous	-	ORF1ab/nsp1
A1005G	6.74	nonsynonymous	K247R, K67R	ORF1ab/nsp2
C1059T	99.87	nonsynonymous	T265I, T85I	ORF1ab/nsp2
T1927C	2.56	synonymous	-	ORF1ab/nsp2
C2096T	16.76	nonsynonymous	Q611 *, Q431 *	ORF1ab, nsp2
C2110T	16.76	synonymous	-	ORF1ab, nsp2
C2523T	98.52	nonsynonymous	T753I, T573I	ORF1ab, nsp2
A4197G	95.31	nonsynonymous	E1311G, E493G	ORF1ab, nsp3
G4257T	3.62	nonsynonymous	G1331V, G513V	ORF1ab, nsp3
G6116C	18.85	nonsynonymous	A1951P, A1133P	ORF1ab, nsp3
C6568T	10.31	synonymous	-	ORF1ab, nsp3
C7691T	15.29	nonsynonymous	Q2476 *, Q1658 *	ORF1ab, nsp3
G7954T	94.57	nonsynonymous	Q2563H, Q1754H	ORF1ab, nsp3
G8999T	25.39	nonsynonymous	A2912S, A149S	ORF1ab, nsp4
C9551G	4.46	nonsynonymous	P3096A, P333A	ORF1ab, nsp4
C9712T	6.46	synonymous	-	ORF1ab, nsp4
A10552G	75.56	synonymous	-	ORF1ab, nsp5
C10718T	5.37	nonsynonymous	R3485 *, R222 *	ORF1ab, nsp5
C10854T	6.33	nonsynonymous	S3530L, S267L	ORF1ab, nsp5
C10965T	6.86	nonsynonymous	T3567I, T304I	ORF1ab, nsp5
G10986T	9.16	nonsynonymous	R3574I, R5I	ORF1ab, nsp6
G11625T	5.95	nonsynonymous	G3787V, G218V	ORF1ab, nsp6
C11668T	5.16		-	ORF1ab, nsp6
T12009C	4.28	nonsynonymous	L3915P, L56P	ORF1ab, nsp7
C12194T	7.90	nonsynonymous	L3977F, L35F	ORF1ab, nsp8
G12692C	6.92	nonsynonymous	E4143Q, E3Q	ORF1ab, nsp9
C13164T	9.25	nonsynonymous	T4300I, T47I	ORF1ab, nsp10
C13274T	7.53	nonsynonymous	P4337S, P84S	ORF1ab, nsp10
C13684T	8.29	nonsynonymous	H4474Y, H82Y	ORF1ab, nsp12
C14325T	8.23	synonymous	-	ORF1ab, nsp12
C16792G	7.61	nonsynonymous	R5510G, R186G	ORF1ab, nsp13
C17452T	9.55	nonsynonymous	P5730S, P406S	ORF1ab, nsp13
G18074T	13.68	nonsynonymous	S5937I, S12I	ORF1ab, nsp14
C23053T	96.43	synonymous	-	s
C23556T	12.38	nonsynonymous	P665L	s
C23625T	17.46	nonsynonymous	A688V	s
C23692T	17.58	synonymous	-	s
G24858T	6.83	nonsynonymous	G1099V	s
C24909T	6.82	nonsynonymous	T1116I	s
G25563T	99.87	nonsynonymous	Q57H	ORF3a
C26882T	6.60	synonymous	-	m
C27196T	11.62	-	-	noncoding region
T27206C	12.23	nonsynonymous	F2S	ORF6
C27389T	20.09	-	-	noncoding region
C27813T	5.64	nonsynonymous	L20F	ORF7
C27964T	2.85	nonsynonymous	S24L	ORF8
G28089T	2.11	nonsynonymous	G66C	ORF8
C28435T	7.78	synonymous	-	n
G28451C	98.29	nonsynonymous	G60R	n
A28715T	98.05	nonsynonymous	T148S	n
C29226T	4.79	nonsynonymous	S318L	n
G29239T	7.06	nonsynonymous	M322I	n

SNV = single nucleotide variation; AA = amino acid; * = premature stop codon.

**Table 3:** Five SNVs unique to the day 46 sample.

SNV	Frequency (%)	SNV Type	AA Change	Gene
C1348T	3.42	synonymous	-	ORF1ab, nsp2
C21530G	2.25	nonsynonymous	S7089C, S291C	ORF1ab, nsp16
G21535T	2.25	nonsynonymous	D7091Y, D293Y	ORF1ab, nsp16
T21534A	2.26	nonsynonymous	S7090R, S292R	ORF1ab, nsp16
A21536C	2.24	nonsynonymous	D7091A, D293A	ORF1ab, nsp16

SNV = single nucleotide variation; AA = amino acid.

**Table 4:** Twenty-five SNVs unique to the day 49 sample.

SNV	Frequency (%)	SNV Type	AA Change	Gene
C683T	8.42	synonymous	-	ORF1ab, nsp1
G1289A	3.79	nonsynonymous	E342K, E162K	ORF1ab, nsp2
C2710T	10.92	synonymous	-	ORF1ab, nsp2
G3483A	16.07	nonsynonymous	G1073E, G255E	ORF1ab, nsp3
G5397C	35.07	nonsynonymous	C1711S, C893S	ORF1ab, nsp3
A5939T	48.59	nonsynonymous	I1892F, I1074F	ORF1ab, nsp3
T7361G	9.64	nonsynonymous	W2366G, W1548G	ORF1ab, nsp3
T8060C	12.93	nonsynonymous	S2599P, S1781P	ORF1ab, nsp3
G8368T	15.97	synonymous	-	ORF1ab, nsp3
C12403T	48.55	synonymous	-	ORF1ab, nsp8
C15701A	16.09	nonsynonymous	S5146 *, S754 *	ORF1ab, nsp12
G15907A	41.67	nonsynonymous	G5215S, G823S	ORF1ab, nsp12
T20961C	7.16	synonymous	-	ORF1ab, nsp16
T21060C	9.13	synonymous	-	ORF1ab, nsp16
G22203T	8.86	nonsynonymous	R214L	s
A22810G	11.06	synonymous	-	s
C23376T	14.03	nonsynonymous	S605F	s
C23816T	5.19	nonsynonymous	L752F	s
A24644G	8.76	nonsynonymous	K1028E	s
T27402C	11.10	synonymous	-	ORF7a
G27621A	7.30	synonymous	-	ORF7a
T27837C	8.08	nonsynonymous	F28L	ORF7b
C28896T	2.75	nonsynonymous	A208V	n
A29469T	4.50	nonsynonymous	D399V	n
G29494T	2.38	nonsynonymous	L407F	n

SNV = single nucleotide variation; AA = amino acid; * = premature stop codon.

## Data Availability

Viral genome data are available in NCBI GenBank, accessions MW729373-MW729375, and raw sequencing reads are available in SRA, accessions SRR17073935- SRR17073938.

## Supplementary Materials

The following are available online at https://www.mdpi.com/article/10.3390/pathogens10121589/s1, Table S1: Acknowledgement table for sequences used in phylogenetic tree, Table S2: Summary of genome sequence data produced in this study and associated accession numbers.

## Disclaimer

The views expressed in this article are those of the authors and do not necessarily reflect the official policy or position of the Department of Defense, the Navy, or the U.S. Government. Several of the authors are U.S. Government employees. This work was prepared as part of their official duties. Title 17 U.S.C. § 105 provides that ‘Copyright protection under this title is not available for any work of the United States Government.’ Title 17 U.S.C. §101 defines a U.S. Government work as a work prepared by a military service member or employee of the U.S. Government as part of that person’s official duties.
