# Supplementary material for: Immunological and Genetic Investigation of SARS-CoV-2 Reinfection in an Otherwise Healthy, Young Marine Recruit
Source: Pathogens. 2021 Dec 8;10(12):1589. doi: 10.3390/pathogens10121589 (PMC8709254; doi:10.3390/pathogens10121589)
Supplement: Supplementary file 1 [file pathogens-10-01589-s001.zip › Table S1.pdf]

Table S1. Acknowledgement table for sequences used in phylogenetic tree.

Accession ID	Originating laboratory	Submitting laboratory	Authors
EPI_ISL_410045	IL Department of Public Health Chicago Laboratory	Pathogen Discovery, Respiratory Viruses Branch, Division of Viral Diseases, Centers for Disease Control and Prevention	Authors Anna Uehara; Brett L. Whitaker; Brian Lynch; Clinton R. Paden; Janna' R. Murray; Jing Zhang; Krista Queen; Lijuan Wang; Senthil Kumar K. Sakthivel; Shifaq Kamili; Stephen Lindstrom; Susan I. Gerber; Suxiang Tong; Xiaoyan Lu; Yan Li; Ying Tao
EPI_ISL_596982	Lighthouse Lab in Cambridge	Wellcome Sanger institute for the COVID-19 Genomics UK (COG-UK) consortium	Cordelia Langford; David K. Jackson; Dominic Kwiatkowski; Ewan Harrison; Ian Johnston; John Sillitoe on behalf of the Wellcome Sanger Institute COVID-19 Surveillance Team (http://www.sanger.ac.uk/covid-team); Rob Howes; Roberto Amato; Sonia Goncalves; The Lighthouse Lab in Cambridge and Alex Alderton
EPI_ISL_577572	Michigan Department of Health and Human services, Bureau of Laboratories	Michigan Department of Health and Human Services, Bureau of Laboratories	Blankenship HM; Riner D; Soehnlen MK
EPI_ISL_6638430	Missouri State Public Health Laboratory	Missouri State Public Health Laboratory	Ashley New; Joshua Barry; Matthew Sinn
EPI_ISL_6661896	Molecular Biology Laboratory, Westchester Medical Center	Genomics Core Laboratory, New York Medical College	Brahmaraju Mopidevi; Humayun K. Islam; Jian Zhuge; Salomon Amar; Utsav Pandey; Weihua Huang
EPI_ISL_660629	NHLS-IALCH	KRISP, KZN Research Innovation and Sequencing Platform	Giandhari J; Khan S; Lessells R; Mdlalose K; Pillay S; Tegally H; Wilkinson E; York D; de Oliveira T
EPI_ISL_1624280	Naval Infectious Diseases Diagnostic Laboratory	Naval Medical Research Center Biological Defense Research Directorate	Andrew Bennett; Bishwo Adhikari; Catherine Arnold; Francisco Malgon Bautista; Gregory Rice; Kimberly Bishop-Lilly; Kyle Long; Lindsay Glang; Logan Voegtly; Megan Schilling; Regina Cer; Victor Sugiharto
EPI_ISL_6782022	State of New Hampshire Public Health Laboratories	State of New Hampshire Public Health Laboratories	Caitlin Mercier; Chris Benton; Jinfeng Li; Juan Bolanos; Xinglu Zhang
EPI_ISL_695544	TGen North	TGen North	Ashlyn Pfeiffer; Chris French; Darrin Lemmer; Dave Engelthaler; Hayley Yaglom; Jolene Bowers; Megan Folkerts; The Arizona COVID Genomics Union (ACGU)
EPI_ISL_572301	Virginia DCLS	Virginia DCLS	Virginia DCLS
EPI_ISL_402124	Wuhan Jinyintan Hospital	Wuhan Institute of Virology, Chinese Academy of Sciences	Ding-Yu Zhang; Hao-Rui Si; Lei Zhang; Peng Zhou; Xing-Lou Yang; Yan Zhu; Zhengli Shi
