# Supplementary material for: Immunological and Genetic Investigation of SARS-CoV-2 Reinfection in an Otherwise Healthy, Young Marine Recruit
Source: Pathogens. 2021 Dec 8;10(12):1589. doi: 10.3390/pathogens10121589 (PMC8709254; doi:10.3390/pathogens10121589)
Supplement: Supplementary file 1 [file pathogens-10-01589-s001.zip › Table S2.pdf]

Table S2. Summary of genome sequence data produced in this study and associated accession numbers.

Sample name	Pango lineage/Clade	GenBank Accession	GISAID Accession	SRA Accession
SARS_CoV_2_CHARM_1167_d7_2020	B.1.340/20C	MW729373	EPI_ISL_6913902	SRR17073938
SARS_CoV_2_CHARM_1167_d14_2020	No lineage assigned/20B	OL678543	EPI_ISL_6913905	SRR17073937
SARS_CoV_2_CHARM_1167_d46_2020	B.1.1/20B	MW729374	EPI_ISL_6913903	SRR17073936
SARS_CoV_2_CHARM_1167_d49_2020	B.1.1/20B	MW729375	EPI_ISL_6913904	SRR17073935
